# Computer-assisted cognitive rehabilitation in neurological patients: state-of-art and future perspectives

**DOI:** 10.3389/fneur.2023.1255319

**Published:** 2023-09-29

**Authors:** Maria Grazia Maggio, Daniela De Bartolo, Rocco Salvatore Calabrò, Irene Ciancarelli, Antonio Cerasa, Paolo Tonin, Fulvia Di Iulio, Stefano Paolucci, Gabriella Antonucci, Giovanni Morone, Marco Iosa

**Affiliations:** ^1^IRCCS Centro Neurolesi “Bonino Pulejo”, Messina, Italy; ^2^Department of Human Movement Sciences, Faculty of Behavioral and Movement Sciences, Amsterdam Movement Sciences & Institute for Brain and Behavior Amsterdam, Vrije Universiteit Amsterdam, Amsterdam, Netherlands; ^3^IRCCS Santa Lucia Foundation, Rome, Italy; ^4^Department of Life, Health and Environmental Sciences, University of L’Aquila, L’Aquila, Italy; ^5^Institute for Biomedical Research and Innovation (IRIB), National Research Council of Italy, Messina, Italy; ^6^S’Anna Institute, Crotone, Italy; ^7^Pharmaco Technology Documentation and Transfer Unit, Preclinical and Translational Pharmacology, Department of Pharmacy, Health Science and Nutrition, University of Calabria, Rende, Italy; ^8^Department of Psychology, Sapienza University of Rome, Rome, Italy; ^9^San Raffaele Institute of Sulmona, Sulmona, Italy

**Keywords:** rehabilitation software, telerehabilitation, cognitive rehabilitation, computer-based, rehabilitation, executive functions, memory, attention

## Abstract

**Background and aim:**

Advances in computing technology enabled researchers and clinicians to exploit technological devices for cognitive training and rehabilitation interventions. This expert review aims to describe the available software and device used for cognitive training or rehabilitation interventions of patients with neurological disorders.

**Methods:**

A scoping review was carried out to analyze commercial devices/software for computerized cognitive training (CCT) in terms of feasibility and efficacy in both clinical and home settings. Several cognitive domains responding to the different patients’ needs are covered.

**Results:**

This review showed that cognitive training for patients with neurological diseases is largely covered by several devices that are widely used and validated in the hospital setting but with few translations to remote/home applications. It has been demonstrated that technology and software-based devices are potential and valuable tools to administer remotely cognitive rehabilitation with accessible costs.

**Conclusion:**

According to our results, CCT entails the possibility to continue cognitive training also in different settings, such as home, which is a significant breakthrough for the improvement of community care. Other possible areas of use should be the increase in the amount of cognitive therapy in the free time during the hospital stay.

## Highlights

- Devices and software for cognitive rehabilitation are a feasible solution with increasing attention also thanks to the distancing of the COVID-19 pandemic.
- With these devices, different cognitive domains can be trained in the hospital or at home.
- These devices and software can guarantee continuity of care between hospital and home even though the same user interfaces.
- It is necessary to overcome problems of various kinds that limit the spread of these devices: geographical and socio-economic barriers.

## 1. Introduction

In recent years, the aging of population in the industrialized countries increased the demand for care services, including neurorehabilitation (1, 2). Then, the overload of healthcare systems and the difficulties in organizing services have required the implementation of new methodologies for rehabilitation (2). COVID-19 has affected rehabilitation processes, especially in neurological patients, harming the quality of life of both patients and their families. To face this unexpected pandemic, new innovative models of rehabilitation service have emerged. At the same time, it led to the increase of non-hospital services to guarantee the continuity of care, thanks to technological innovations (3). Moreover, rapid advances in computing technology have enabled researchers to carry out cognitive training and rehabilitation interventions with the assistance of technology (4).

Cognitive rehabilitation (CR) aims to improve residual neuropsychological capacities through specific strategies based on cognitive models. In particular, the innovative techniques mediated by personal computers (PC) use multimedia and computer resources, through hardware and software systems, to implement cognitive functioning, including attention, memory, problem-solving, language, and executive functions (5–8). Computerized methods are based on repeated training of specific cognitive domains, through the execution of tasks involving specific skills. Most of the tools use audio-video feedback as a motivational stimulus. Furthermore, these tools allow modifying the type, duration, and difficulty of the tasks to adapt the intervention to the individual abilities. The exercises are grouped according to the cognitive domain stimulated and adapt to the patient’s abilities to avoid frustration due to too complex or too simple tasks (5). These devices could offer some therapeutic possibilities for the CR of various neurological diseases (4–8). It has been shown that innovative tools, such as PC-based treatments, could facilitate patient management in the rehabilitation process, allowing continuity of care at home through the telerehabilitation mode (9–13). The tools could support restorative training on cognitive functions thanks to the simulation of different cognitive domains, with positive repercussions on the patient’s motivation (4–8). Various authors have also shown that telerehabilitation can improve various cognitive domains, with results comparable to those of conventional face-to-face rehabilitation (3, 10). Despite the many advantages of PC-based approaches, these devices have also some limitations such as: (a) visual interface limiting their use; (b) access prerequisites (i.e., computer skills); (c) lack of acceptability due to photosensitivity problems; (d) acceptability of devices; (e) reliability (some systems have been validated for the clinic but not remotely, or not validated on some types of the population); (f) availability (some systems are too expensive for a patient to maintain or purchase). However, there are neither clear indications nor warnings for the use of these tools, as rigorous comparisons of technical devices in different neurological populations have never been performed.

In this review, we sought to provide an overall picture of the devices on the market that can be used for CR. Furthermore, a secondary aim is to report on the strengths and weaknesses of the devices employed in inpatient or remote/home settings for continuing the rehabilitation process.

## 2. Search strategy

This scoping review was conducted by searching peer-reviewed articles published between 01 June 2010 and 31 December 2022 using the following databases: PubMed, Embase, Cochrane Database, and Web of Science. The aim of the search strategy was twofold: (1) to track progress in the use of software and device-based technology in terms of technological content, human-machine interaction, and cognitive domain training, and (2) to check neurological populations in which the devices and software for cognitive neurorehabilitation are used. To this end, a comprehensive search was carried out using the search terms: (“Cognitive Rehabilitation” OR “Computer-based” OR “Telerehabilitation”) AND (“Stroke” OR “Traumatic Brain Injury” OR “Dementia” OR Multiple Sclerosis” OR “Parkinson” OR “Rehabilitation”). After the removal of the duplicates, all articles were evaluated based on the titles and abstracts. The inclusion criteria were: (i) patients with neurological disease; (ii) a computerized approach applied to cognitive rehabilitation; (iii) English language; and (v) published in a peer-reviewed journal. We excluded articles that described theoretical models, methodological approaches, algorithms, basic technical descriptions, and validation of experimental devices providing no clear translation to clinical practice. Furthermore, we excluded: (i) animal studies; (ii) conference proceedings, or reviews; (iii) studies focusing only on other innovative approaches (such as virtual reality, exergaming, or serious games), (iv) cognitive remediation relating to physiological condition (i.e., the developmental stage or the elderly), (v) study concerning the mobile-app device, which is too far from the traditional CR program.

The list of articles was then refined based on relevance and summarized according to the inclusion/exclusion criteria. Furthermore, to ensure a greater homogeneity in the results, after the removal of duplicates, the articles were evaluated on the basis of the titles and abstracts by two independent researchers (DDB and MGM). These researchers read the full text of articles suitable for the study and performed the data collection to reduce the risk of bias (i.e., language bias; publication bias; time-lag bias). In case of disagreement on the inclusion and exclusion criteria, the final decision was made by two senior investigators (RSC and GM).

**Figure 1:**
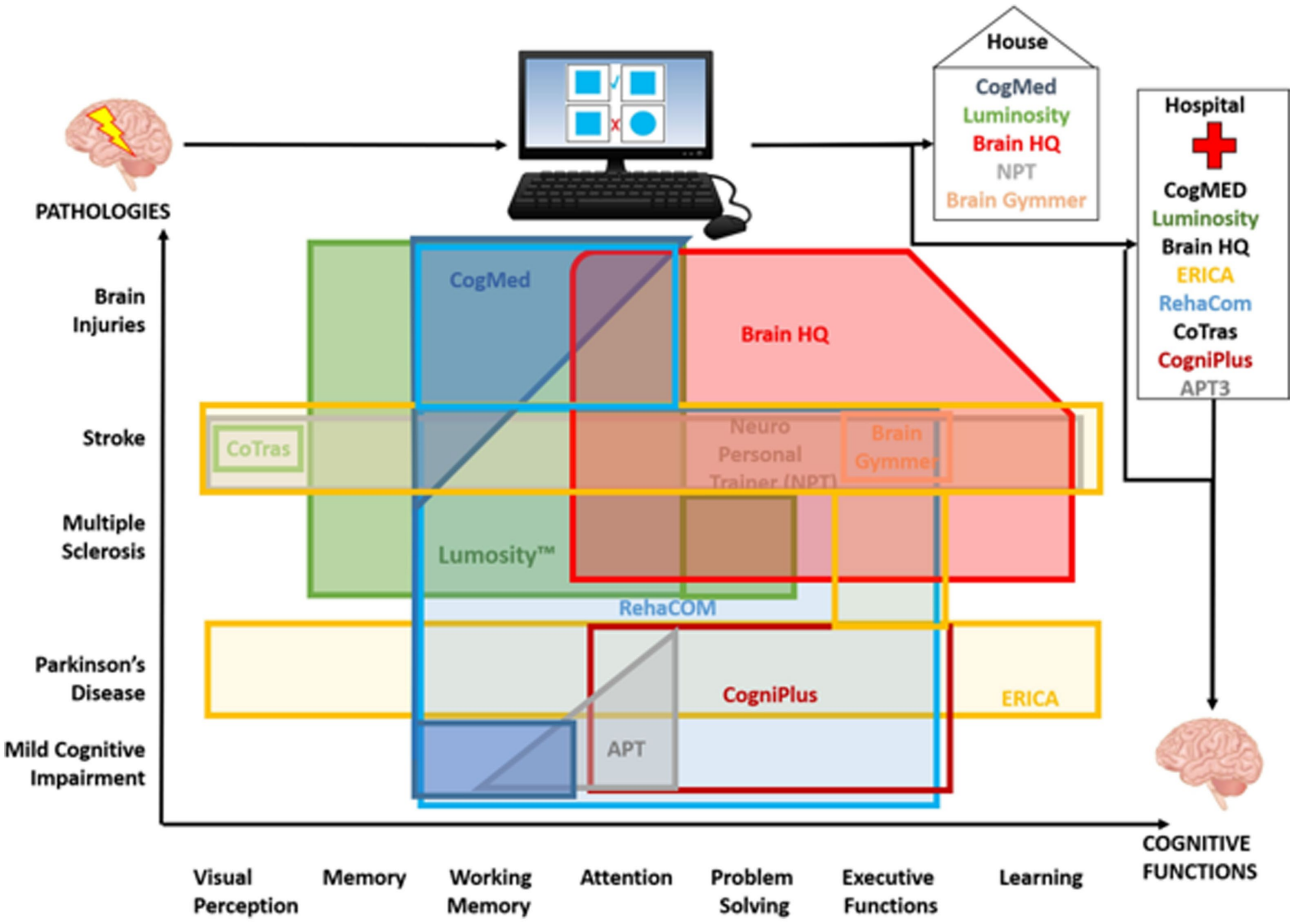
Schematic representation of the clinical use of the different cognitive devices and software. The figure illustrates the different devices and cognitive functions trained in the different pathologies and in which setting they were used (home or in hospital). The figure reflects the fields of application as evident from the literature selected in this review.

Data extraction was performed on 190 articles. Data were considered for the following information: year and type of publication (e.g., clinical studies, pilot study), characteristics of the participants involved in the study, and purpose of the study (Figure 1). After a thorough review of the complete manuscripts, 34 studies articles met the exclusion/exclusion criteria (Table 1). We reported as a primary outcome the one identified by the researchers, for each study, as between-group (in RCT design) or within-subjects difference (for studies with only one group) on the first-level test, and secondary outcome as differences within groups (for RCT) or on second-level tests (for single-group studies). For every study, we selected only significant results adjusted for multiple comparisons.

**Table 1:** List of devices and their main characteristics, main studies, and clinical populations on which they have been tested.

Device	Device description	License	Type of feedback	Adaptive training approach	Studies	Neurologic population	Cognitive domains
CogMED QM training, Pearson Company, Stockholm, Sweden, 2011 (www.cogmed.com) Type: Software	The CogMed is a computer-based solutions software for cognitive training of attention problems caused by poor working memory (WM). Use of Cogmed requiringes a computer and/or tablet with speakers, stable broadband internet connection of 0.5 Mbit/s or higher, Adobe Flash plugin version 10.0 or later, and minimal hard drive space to store results. It is programmed for Mac, PC, and Android devices, but. It is most commonly run online through the Cogmed website where users are provided a unique ID and password.	Professionals use only. Available on subscription	Coach online	Available	Akerlund et al. (2013) (14)	ABI	Working Memory
					Johansson & Tornmalm (2012) (15)	ABI	Working Memory, Activity of Daily Living (ADL)
					Lundqvist et al. (2010) (16)	ABI	Spatial and Verbal Working Memory
					Svaerke et al. (2022) (17)	ABI	Working Memory
					Blair et al. (2021) (18)	MS	Spatial and Verbal Working Memory
					Nyberg et al. (2018) (19)	Stroke	Working Memory
Lumosity™ Brain Games Lumos Labs. Lumosity: Reclaim Your Brain™. San Francisco, CA: Dakim, Inc.; 2010 (www.lumosity.com) Type: Web platform/software app	LumosityTM is a commercially available CCT software providing brain games designed to improve cognitive processing speed, flexibility, attention, memory, and problem-solving skills. Game complexity increases and decreases systematically based on an individual’s performance data. Multiple forms of each game level are available to prevent task learning with continued practice.	Full version available on payment. Free- version mode (with a smaller number of exercises chosen randomly)	Real-time feedback on errors; Lumosity performance index at the end of each exercises	Available	Withiel et al. (2019) (20)	Stroke	Everyday Memory
					Wentink et al. (2016) (21)	Stroke	Working Memory, Attention, Fluency, Quality of Life
					Stuifbergen et al. (2018) (22)	MS	Memory, Attention, Problem Solving Skills
					Zickefoose et al. (2013) (23)	TBI	Attention
BrainHQ program, Posit Science Corporation, San Francisco CA, 2015 (www.brainhq.com) Type: Web platform/software app	BrainHQ is a software designed for brain stimulation of: Attention, Brain Speed, Memory, People Skills, Intelligence, and Navigation. It is an adaptive software, easy to use, and can be implemented on home computers, tablets, and laptops. Based on scientific research results, it allows choosing different levels/types of training based on individual needs.	Full version available on payment. Free- version mode (with a smaller number of exercises chosen randomly)	Graphic and numeric feedback regarding patients’ performance within and across sessions per game and category of cognitive function, adjusted to matched demographics	Available	Yeh et al. (2019) (24)	Stroke	Attention, Executive Functions
					Charvet et al. (2017) (25)	MS	Attention, Information processing, Learning
					O’Neil-Pirozzi & Henry Hsu (2016) (26)	TBI	Attention, Memory, Executive Functions
NeuroPersonalTrainer”®, GNPT®^,^ Guttmann Institute, Badalona, Spain, 2011. Type: Tele-rehabilitation platform	GNPT® allows the provision of individualized and personalized treatments, improving the traditional on-site rehabilitation processes. It is based on a testing and training approach, so after a first baseline the software select specific training and store patients’ scores by organizing them in a therapeutic index on which next exercises will be adjusted.	Professionals use only. No free trial available	The program will then calculate a cognitive profile using these results, taking into account the patient’s age and educational level.	Available	Gil-Pages et al. (2018) (27)	Stroke	Multiple domains.
ERICA, Giunti Psycometrics, Italy, 2013 (www.giuntipsy.it) Type: Software	Erica is software for customized cognitive rehabilitation involving 5 specific cognitive domains: attention process, memory abilities, spatial cognition, verbal and nonverbal executive functions. The training is characterized by modularity, flexibility, and uniformity of the administered program.	Professionals use only. Three years license based on subscription.		Not available automatically, therapist may adjust the preferred exercises based on patients’ performance	De Luca et al. (2019) (28)	Parkinson’s Disease (PD)	Multiple domains
					De Luca et al. (2017) (29)	Stroke	Multiple domains
					Barbarulo et al. (2018) (30)	MS	Executive Functions.
CogniPlus, Schuhfried GmbH, Vienna, Austria, 2008 (www.schuhfried.com) Type: Software	CogniPlus software has various modules for the training of specific cognitive abilities. The content of CogniPlus is closely linked to the Vienna Test System. This software combines treatment and evaluation exploiting the role of the therapeutic assessment CogniPlus is an intelligent interactive system that adapts to patients’ abilities by offering exercises based on performance.	Professionals use only. No free trial available.	Feedback is provided by testing patient’s ability. This software combines a testing and training approach	Available	Hagovská et al. (2017) (31)	older adults with MCI	Multiple domains
					Zimmermann et al. (2014) (32)	Parkinson’s Disease (PD)	Executive Functions, Focused Attention.
Attention Process Training (APT), Lash & Associates Publishing/Training Inc., Youngsville, North Carolina, 2010 (https://lapublishing.com). Type: Software	APT is software for cognitive rehabilitation of attention abilities, based on structured exercises for training specific cognitive domains of attention. A graphical interface allows the clinician to select exercises and associated parameters in order to create customized exercise easily modifiable as the patient progresses.	Full version available on payment. The software is licensed for a period of one year.	Feedback is provided as scores on each exercise at the end of session.	Not reported	Pantoni et al. (2017) (33)	MCI	Attention, working memory
					Walton et al. (2018) (34)	Parkinson’s Disease (PD)	Attention, ADL
CoTras, RPIO Co., Ltd., Geumcheon-gu, Seoul, 2010 (www.rpio.co.kr). Type: Software with specific hardware for human-computer interaction	CoTras is a training program involving visual perception, attention, memory, orientation, and others (categorization, sequencing). A joystick and a large button on the CoTras panel make the training easy for patients who are unfamiliar with computer use.	Professionals use only. No free trial available.	Feedback is provided at the end of session with graphs and statistic report about patient’s performance.	Available	Park and Park (2015) (35)	Stroke	Visual Perception
BrainGymmer, Dezzel Media, The Netherlands, 2010 (www.braingymmer.com) Type: Web platform	BrainGymmer is an online brain training program that offers brain games, brain tests, and brain teasers. It has been developed and clinically tested by clinicians and academics. Individual performance is further compared to people of the same age	Full version available on payment. Free- version mode (with a smaller number of exercises chosen randomly).	Feedback is provided at the end of session based on the Brain Fitness Index (accuracy and reaction speed of the performance)	Not reported	Van de Ven et al. (2017) (36)	Stroke	Executive Functions
RehaCom®, HASOMED GmbH, Magdeburg, Germany, 1997. www.rehacom.com Type: Software with specific tool for human-computer interaction.	RehaCom is a cognitive rehabilitation program consists of 20 modules with several subsections that are selected and used by the therapist according to the needs of the participant. The RehaCom hardware has a special keyboard with large buttons, which limits the interference of motor and coordination impairment and expertise in computer use. Online monitoring is also available for the therapist to assess the function of the participant.	Professionals use only. No free trial available.	Progress can be saved, but no direct feedback is provided at the end of exercises.	Available	Nousia et al. (2022) (37)	MCI	Multiple Domains
					Naeeni Davarani et al. (2022) (38)	Multiple Sclerosis (MS)	Attention, executive functions, working memory
					Amiri et al. (2021) (39)	Stroke	Working Memory, Processing Speed
					Messinis et al. (2020) (40)	MS	Multiple Domains
					Messinis et al. (2017) (41)	MS	Multiple Domains
					Campbell et al. (2016) (42)	MS	Attention, Processing Speed
					Bonavita et al. (2015) (43)	MS	Attention
					Darestani et al. (2020) (44)	MS	Verbal performance
					Veisi-Pirkoohi et al. (2020) (45)	Stroke	ADL, attention
					Fernandez et al. (2017) (46)	ABI	Attention, memory
					Cerasa et al. (2014) (47)	PD	Attention, Information Processing
GRADIOR (INTRAS Foundation, Spain) Type: Software	GRADIOR is multimedia software for cognitive stimulation, neuropsychological assessment, and rehabilitation. It consists of personalized exercises that train various cognitive domains, such as attention, memory, orientation, calculation, perception, reasoning, and language.	Professionals use only.	The evaluation profile generated by the program offers a description of cognitive performance	Not Reported	Diaz Baquero et al. (2022) (8)	MCI and mild Dementia	Executive functioning, attention, phonological verbal fluency, cognitive flexibility
					Diaz Baquero et al. (2022) (48)	MCI and mild Dementia	Executive functioning, attention, phonological verbal fluency, cognitive flexibility
					Góngora Alonso et al. (2020) (49)	Severe and prolonged mental illness	Psychosocial skills
					Vanova et al. (2018) (50)	MCI dementia	Cognition, mood, quality of life, activities of daily living, quality of patient-carer relationship

ABI, Acquired Brain Injury; EG, Experimental group; CG, Control group; MCI, Mild Cognitive Impairments; MS, Multiple Sclerosis; PD, Parkinson’s Disease; TBI, Traumatic Brain Injury.

## 3. Results

Although our research in PubMed, Embase, Cochrane Database, and Web of Science has found many technological devices used in CR, only the 10 most cited devices were selected. The information obtained from the selection of studies was organized in two tables. Table 1 reports the list of devices and their main characteristics, as well as the studies and clinical populations on which they were tested. Table 2, indeed, shows what type of study was carried out, how the device was used, and what the results are in terms of treatment efficacy. Most of the selected devices (6 out 11) are supplied in software mode. Then, they can be used by purchasing a stand-alone license, which has a limited duration to the subscription chosen on the manufacturer’s website. Of these, only CogMED (14–19) provides a special license for its usage and a specific training for the online tutor. Three devices (Lumosity, Brain HQ, Brain Gymmer) are either available as PC software or can be installed as an app on tablet/phone devices. Finally, only one represents a telerehabilitation platform (NeuroPersonalTrainer) that allows patients to carry out Hospital and home rehabilitation. In most studies included in Table 2, these devices were tested to evaluate their effectiveness compared to conventional rehabilitation. This review reveals that cognitive training for patients with neurological diseases is covered by several efficient devices that are widely used and validated in the hospital setting, but with few translations in remote applications.

**Table 2:** Performance results reported in the selected studies of this review.

Device	Studies	Sample characteristic	Type of study	Training protocols	Treatment duration location supervision	Outcome measures & efficacy
CogMED QM training, Pearson Company, Stockholm, 2011 Sweden, (www.cogmed.com).	Akerlund et al. (2013) (14)	38 Acquired Brain Injury (18 CG)	RCT	Both groups underwent integrated rehabilitation. The experimental group also implemented the computerized training program Cogmed	5-week training program (30–45 min, 5 days). In Hospital Supervision of a therapist	BNIS (*p_b_* = 0.044), Digit Span (*p_b_* = 0.045), Digit Span reverse (*p_b_* = 0.003)
	Johansson & Tornmalm (2012) (15)	18 chronic stage patients with ABI: traumatic brain injury (5), brain tumor (6), stroke (7) (severe impairment)	Cross-over study with no CG	Customize training program	7–8 weeks (20–25 sessions) 30–45 min in Hospital Group of 5–6 participants, under supervision of a therapist	QM index (*p_w_* = 0.000), Cognitive Failures Questionnaire (*p_w_* = 0.018), Canadian Occupational Performance Measure (*p_w_* = 0.008)
	Lundqvist et al. (2010) (16)	21 chronic stage patients with ABI: stroke (1), trauma (11), infection (5), tumor (2), subarachnoidal hemorrhage (2) (mild to moderate neurologic disorder).	Cross-over study with no CG	Remember the position of stimuli in a four-by-four grid, reproduce stimuli order, remember sequences of letters and digits forwards and/or backward.	5 weeks (25 sessions) 45–60 min In Hospital Under a coach supervision	PASAT (*p_w_* < 0.001), Listening Span (*p_w_* < 0.001), Block Span forward (*p_w_* = 0.002), backward (*p_w_* = 0.001)
	Svaerke et al. (2022) (17)	72 patients with ABI	RCT	Treatment was diversified into four different groups: two groups trained with the “Cogmed” and “Brain + Health” programs, respectively, and one group completed active control training. All three groups received ongoing support from a health professional. The last group trained under the ‘Brain+ Health’ program but received no support	12-week intervention. In Hospital Supervision of a therapist	Both CBCR programs improved working memory when administered with support from a health professional. The programs have improved several subcomponents of working memory.
	Blair et al. (2021) (18)	22 chronic stage patients with MS (moderate impairment) (CG:11)	Single blind RCT	Remember the position of stimuli in a four-by-four grid, reproduce stimuli order, remember sequences of letters and digits forwards and/or backward. CG patients received a TAU.	5 weeks (25 sessions) 30–45 min At home Coaching online	DKEFS (*p_w_* = 0.02) Colour-Word Interference test (*p_w_* = 0.016), Digit span (*p_w_* = 0.01).
	Nyberg et al. (2018) (19)	22 chronic stage patients with Stroke (mild impairment)	Cross-over study with no CG	“Grid” (visuospatial working memory); “Numbers” (verbal and visuospatial working memory); “Cube” (visuospatial working memory) and “Hidden numbers” (verbal working memory)	5 weeks (25 sessions) 40 min training In Hospital Individual feedback once a week	Performance improvement for WAIS subtest Grid, Numbers, Cube, Hidden numbers (*p* < 0.001), no FA changes.
Lumosity™ Brain Games Lumos Labs. Lumosity: Reclaim Your Brain™. San Francisco, CA: Dakim, Inc.; 2010. (www.lumosity.com)	Withiel et al. (2019) (20)	65 chronic stage patients with Stroke (mild impairment).	3-group, single-blind RCT	Recall semantic information (names, conversations, birthday dates); prospective memory tasks. (two control groups, MSG group followed a TAU for memory skills (24), the WC group (19) did not any add-on rehab activity)	6 weeks (30 sessions) 120 min training At Home Weekly update with a therapist	Goal Attainment Scores (*p_b_* = 0.00), Verbal WM (*p_b_* < 0.05) and Prospective memory (*p_b_* < 0.01) for MSG group
	Wentink et al. (2016) (21)	107 stroke patients (GC 57)	RCT	The intervention consisted of a brain training program (Lumosity Inc.®). The control group received general information about the brain week	8-week (24 sessions) At home Not specified	TMT-B, Time B, (*p_b_* = 0.04) and flexibility (TMT-A/TMT-B), Difference time A and time B, (*p_b_* < 0 0.01)
	Stuifbergen et al. (2018) (22)	183 chronic stage patients with MS (mild-to-moderate impairment). (CG 90)	RCT	CCT followed the MAPSS-MS approach (Memory, Attention, Problem-Solving Skills in MS). CG patients, performed a generic computerized training	8 weeks (24 sessions) 45 min training At Home Not specified	CVLT delayed (*p_b_* = 0.012), PASAT 3″ (*p_b_* = 0.006), CESD (*p_b_* = 0.006).
	Zickefoose et al. (2013) (23)	4 chronic stage patients with TBI (severe impairment)	Repeated treatment design (A-B-A-C-A).	Five attention-oriented Lumosity games: Birdwatching, Monster Garden, Playing Koi, Rotation Matrix, and Top Chimp.	4 weeks (16 sessions) 30 min training In Hospital with the therapist.	Pre-post analysis of performance level measured in Lumosity (*p*_w_ < 0.001).
BrainHQ program, Posit Science Corporation, San Francisco CA, 2015. (www.brainhq.com)	Yeh et al. (2019) (24)	30 subacute stage patients with Stroke (mild impairment) (GC 15)	Single-blind, multisite RCT	Sequential training (motor aerobic exercises followed by CCT). Computerized cognitive exercises consisted of tasks involving color and shape identification, calculation, visuospatial object recognition. CG received a modified program of aerobic motor activity	12–18 weeks (36 sessions) 30 min training In Hospital Under the supervision of a therapist.	MoCA (*p_b_* = 0.030), Spatial Span (*p_b_* = 0.012), 6MWT (*p_b_* = 0.025).
	Charvet et al. (2017) (25)	135 chronic stage patients with MS (mild impairment) (CG: 61)	Double-blind, with active-placebo RCT	Speed, Attention, Working Memory, and Executive Functions (visual and auditory domains). CG patients followed a nonspecific training with Hoyle Puzzle and Board Games	12 weeks (60 sessions). 60 min training At Home Remote control of compliance and online supervision through WorkTime Software.	Compliance rates (*p_b_* = 0.0056), PASAT 2″ (*p_b_* < 0.05), DKEFS (*p_b_* < 0.05).
	O’Neil-Pirozzi & Henry Hsu (2016) (26)	14 chronic stage patients with TBI (moderate-to-severe impairment) (CG: 7)	Mixed methods design pilot study	Customize exercises program. CG patients received a general computerized training TAU	20 weeks (98 sessions) 60 min training In Hospital Under the supervision of a therapist (with active feedback).	Hopkins Verbal Learning Test-Revised (*p_b_* = 0.0068), TMT A-B (*p_b_* = 0.0761)
NeuroPersonalTrainer”®, GNPT®, Guttmann Institute, Badalona, Spain, 2011.	Gil-Pages et al. (2018) (27)	40 chronic stage patients with Stroke (mild impairment)	Double-blind, crossover RCT with two arms.	Customize exercises program adjusted on NPE.	6 weeks (30 sessions) 60 min training At Home Under remote control of a therapist, (ongoing study)	Study protocol.
ERICA Giunti Psychometrics, Italy, 2013. (www.giuntipsy.it)	De Luca et al. (2019) (28)	60 chronic stage patients with PD (mild-to-moderate) (CG:30)	RCT	Customize exercises program. CG patients received a TAU	8 weeks (24 sessions) 60 min training In Hospital. Under the supervision of a therapist.	ACE-R (*p_b_* = 0.026§), WEIGL Test (*p_b_* = 0.026§), Hamilton Rating Scale (*p_b_* = 0.031§).
	De Luca et al. (2017) (29)	35 subacute stage patients with Stroke (moderate impairment) (CG:15)	RCT	Customize exercises program. CG patients received a general computerized training TAU	8 weeks (24 sessions) 45 min training In Hospital Under the supervision of a therapist.	MMSE (*p_w_* < 0.01), Attentive Matrices (*p_w_* < 0.021), Letter Verbal Fluency (*p_w_* < 0.06), Categorial Verbal Fluency (*p_w_* < 0.03).
	Barbarulo et al. (2018) (30)	63 chronic stage patients with MS (mild-to-moderate impairment). (CG:32)	RCT	Dual-task exercises, plus additional exercises tailored to the single patient’s neuropsychological impairments. CG patients received only motor TAU	24 weeks (48 sessions) 60 min training In Hospital Not specified	Spatial Span (*p_w_* < 0.003), Forward/Backward verbal span (*p_w_* < 0.032, *p_w_* < 0.027), Phonological Fluency (*p_w_* < 0.001), SRT-D (*p_w_* < 0.001§), WLG (*p_w_* = 0.002§), Tinetti scale (*p_w_* < 0.001)
CogniPlus, Schuhfried GmbH, Vienna, Austria, 2008. (www.schuhfried.com)	Hagovská et al. (2017) (31)	60 MCI patients: (CG 30)	RCT	Group A (*n* = 30) underwent CogniPlus, a computer-based, cognitive training. Group B (*n* = 30) underwent classical group-based cognitive training	8 weeks (24 sessions) Not specified	QOL (*p_w_* < 0.001), attention (increased load score), (*p_w_* < 0.05), errors (*p_w_* < 0.001). No group difference.
	Zimmermann et al. (2014) (32)	39 chronic stage patients with PD (moderate impairment) (CG:20)	RCT	Tasks involved four modules: FOCUS, for focused attention; NBACK, for working memory; PLAND, for planning and action skills and HIBIT, for response inhibition. CG patients underwent a cognitive stimulation with Nintendo Wii	4 weeks (12 sessions) In Hospital Under the supervision of a therapist	Attentional Performance Test (*p_w_* = 0.024§), TMT B/A (*p_w_* = 0.431§), Executive function (*p_w_* = 0.462§), WAIS Block Design Test (*p_w_* = 0.055§), e California Verbal Learning Test (*p_w_* = 0.093§).
Attention Process Training (APT), Lash & Associates Publishing/Training Inc., Youngsville, North Carolina, 2010. (https://lapublishing.com).	Pantoni et al. (2017) (33)	43 MCI patients (CG 22)	RCT	CG received the standard care and EG performed the attention training	40h (2-h weekly sessions for 20 weeks) Not specified	Rey Auditory-Verbal Learning Test immediate recall (change score 6 versus 12 months: 1.8 ± 4.9 and − 1.4 ± 3.8, *p* = 0.021; baseline versus 12 months: 3.8 ± 6.1 and 0.2 ± 4.4, *p* = 0.032)
	Walton et al. (2018) (34)	38 chronic stage patients with PD (mild-to-moderate impairment) (CG:18)	Double-blind active RCT	Customize exercises program based on NPE. CG patients received an aspecific cognitive training	7 weeks (14 sessions) In Hospital Group of ten participants, under supervision of a therapist.	Baseline and Follow-Up Geometric Means for the FoG in on-phase (*p_b_* = 0.002).
CoTras, RPIO Co., Ltd., Geumcheon-gu, Seoul, 2010. (www.rpio.co.kr).	Park and Park (2015) (35)	30 subacute stage patients with Stroke (moderate impairment) (CG:15)	RCT	Tasks involving object recognition, object constancy, figure-ground organization, visual discrimination, and visual organization. CG patients received a paper and pencil training for perception rehabilitation	4 weeks (20 sessions) 30 min training In Hospital Under the supervision of a therapist.	Lowenstein Occupational Therapy Cognitive Assessment (*p_b_* < 0.05§), Motor-free Visual Perception Test-3 (*p_b_* < 0.05§)
BrainGymmer, Dezzel Media, The Netherlands, 2010. (www.braingymmer.com)	Van de Ven et al. (2017) (36)	97 subacute-to-chronic stage patients with Stroke (moderate impairment) (CG 59)	Double-blind RCT	Cognitive flexibility tasks (updating, set-shifting, inhibition). CG groups: AC 35 patients followed a mock training, WLC 24 patients received TAU	12 weeks (58 sessions) At Home Under the supervision of a therapist.	TMT-B (*p_w_* < 0.001§), LNS (*p_w_* < 0.01§), ToL (*p_w_* < 0.01§).
RehaCom®, HASOMED GmbH, Magdeburg, Germany, 1997. (www.rehacom.com) Type: Software with specific tool for human-computer interaction.	Nousia et al. (2021) (37)	46 MCI patients (GC 21)	RCT	Multidomain cognitive training intervention program. CG received TAU	30 60-min individual sessions over a period of 15 weeks (i.e., two sessions per week) At home Not specified	Delay memory, Semantic Fluency, TMT-A (*p_b_* < 0.001), Boston Naming Test (*p_b_* = 0.030), Clock Drawing Test (*p_b_* = 0.017), Digit Span Backward (*p_b_* = 0.045) TMT-B (*p_b_* = 0.010)
	Naeeni Davarani et al. (2022) (38)	60 MS patients (CG 30)	RCT	The cognitive trained was focused on: attention, response control, processing speed, working memory, visuospatial skills, and executive functions; CG group received no any intervention,	5 weeks (two 60-min sessions per week)	Visuospatial and motor skills (*p_b_* < 0.01); Verbal executive functions (*p_b_* < 0.01); Non-verbal executive functions (*p_b_* < 0.01); Processing speed (SDMT) (*p_b_* < 0.001); Working memory (*p_b_* < 0.001)
	Amiri et al. (2021) (39)	50 chronic stage patients with Stroke (mild-to-moderate impairment) (CG: 25)	RCT	Customize exercises program. CG patients engaged in routine physiotherapy rehabilitation sessions without any extra cognitive stimulation	5 weeks (10 sessions) 30 min training In Hospital Group of 10 participants, under supervision of a therapist.	N-back (*p_b_* < 0.05), PASAT (*p_b_* < 0.001), SDMT (*p_b_* < 0.001).
	Messinis et al. (2020) (40)	36 MS (CG 17)	Randomized, multi-site, sham controlled trial	Treatment with the RehaCom modules consisted of 24 domain and task specific. The CG completed nonspecific computer based activities	45 min per (3 sessions per week) for 8-week At home Not specified	Verbal learning (*p_b_* < 0.0005), visuospatial memory (*p_b_* < 0.0005) and information processing speed (*p_b_* < 0.0005)
	Messinis et al. (2017) (41)	58 MS patients (CG 26)	RCT	Multidomain computerized treatment, CG received TAU.	10 week (2 days a week for approximately 60 min) At home Not specified	RTLTS (*p_w_* = 0.000), SRTDR (*p_w_* = 0.001), BVMT-R (*p_w_* = 0.001), TMT-A (*p_w_* = 0.000), TMT-B (*p_w_* = 0.000), SNST (*p_w_* = 0.000).
	Campbell et al. (2016) (42)	38 patients with MS (CG 19)	RCT	Treatment sessions consisted of training in three specific modules involving working memory, visuospatial memory, and divided attention. CG watched a series of natural history.	45 min, 3 times weekly for 6 weeks At home Not specified	SDMT (*p_w_* = 0.005)
	Bonavita et al. (2015) (43)	32 chronic stage patients with MS (mild impairment) (CG:14)	RCT	The training program included: “attention and concentration,” “plan a day,” “divided attention,” “reaction behavior,” and “logical thinking” sessions. CG patients received a TAU and an aspecific cognitive stimulation	8 weeks (16 sessions) 50 min training In Hospital Under the supervision of a therapist.	SDMT (*p_w_* = 0.01), PASAT 3″(*p_w_* = 0.00), PASAT 2″ (*p_w_* = 0.03), SRT-D (*p_w_* = 0.02), 10/36 SPART-D (*p_w_* = 0.04), MRI fractal anisotropy (*p_w_* = 0.05).
	Darestani et al. (2020) (44)	60 patients with MS (CG 30)	RCT	Multidomain treatment, CG received no treatment	10 sessions for 5 weeks (2 60 min sessions per week) At home Not specified	CVLT-II (*p* < 0.001) and COWAT (*p* < 0.001)
	Veisi-Pirkoohi et al. (2020) (45)	50 stroke patients (CG 25)	RCT	Multidomain treatment, CG received no treatment	10 sessions (45-min for each) in 5 weeks At home Not specified	ADL, attention and response control (*p_b_* < 0.001)
	Fernandez et al. (2017) (46)	80 ABI patients (GC 30)	RCT	Specific training for attention. CG received TAU	5 session (50 min. For each) per 8 week Not specified	TMT-A, Digit Span and logical memory (*p_b_* < 0.001)
	Cerasa et al. (2014) (47)	20 chronic stage patients with PD (mild impairment) (CG:10)	RCT	Customize exercises program. CG patients performed a simple visuomotor coordination tapping task by using an in-house software	6 weeks (12 sessions) 60 min training In Hospital Group of participants, under supervision of a therapist.	SDMT (*p_b_* = 0.04), Digit Span Forward (*p_b_* = 0.01)
GRADIOR (INTRAS Foundation, Spain)	Diaz Baquero et al. (2022) (8)	43 MCI and mild dementia patients	RCT	computer-based cognitive rehabilitation program and allows CCT on cognitive functions, each of these cognitive modalities includes various sub-modalities to customize the exercises according to the user’s cognitive profile	GRADIOR consisted of attending 2–3 weekly sessions for 4 months with a duration of 30 min	Digit Symbol of WAIS-III (*p* = 0.02), Arithmetic of WAIS-III (*p* = 0.02) and lexical verbal fluency (LVF)-R (*p* = 0.03)
	Diaz Baquero et al. (2022) (48)	Eighty-nine patients with MCI and Dementia (CG = 32)	RCT			Mini-Mental State Exam (MMSE), Trail Making Test (TMT)-A (*p* = 0.03; =0.019)
	Góngora Alonso et al. (2020) (49)	83 subjects with severe and prolonged mental illness	Usability			Gradior has 81.2% acceptance and 83.7% general assessment
	Vanova et al. (2018) (50)	400 people with MCI and mild dementia	RCT		three to four times per week for 30 min per session.	ADASCog Alzheimer’s Disease Assessment Scale – Cognitive Subscale, ADL activities of daily living, CAMCog Cambridge Cognition Examination, EQ5d-5 L EuroQoL 5 dimensions, 5 levels, GDS Geriatric Depression Scale, MMSE Mini Mental State Examination, QCPR Quality of Patient-carer Relationship, SUS System Usability Scale, TMT Trail-making test

Significance is reported as p_w_ (for within groups) and p_b_ (for between groups) value of primary and secondary outcomes measures, with § for significance found in primary outcomes measure also for the control group. For every study, we selected only significant results adjusted for multiple comparisons. MS, multiple sclerosis; PD, Parkinson’s Disease; ABI, acquired brain injury; TBI, Traumatic Brain Injury; RCT, randomized controlled trial; CG, control group; DKEFS, Delis-Kaplan Executive Function System; MRI, magnetic resonance imaging; IQ, Intelligence quotient; WAIS, Wechsler Adult Intelligence Scale; TAU, treatment as usual; WM, working memory; CCT, cognitive computerized training; CVLT, California Verbal Learning test; CESD, Center for Epidemiologic Studies Depression Scale; COWAT, Controlled Oral Word Association Test; MoCA, Montreal Cognitive Assessment; 6MWT, six-minute walking test; TMT A-B, Trial Making Test part A-B; MMSE, Mini-Mental State Examination; ACE-R, Addenbrooke Cognitive Examination Revised; SDMT, Symbol Digit Modalities Test; SPART-D, Spatial Recall Test-Delayed; LNS, Letter Number Sequencing; ToL, Tower of London; FoG, freezing of gait.

### 3.1. CogMED

CogMed (*QM Training, Pearson Company, Stockholm, Sweden, 2011*) is a computer-based software system for training of attention and working memory (WM). CogMed can be accessed via computer and tablet with speakers, and it is mainly used online through the Cogmed website. It has been shown that this device could have positive effects on the rehabilitation of WM. Akerlund et al. carried out a randomized study of 47 patients with acquired brain injury (ABI) in the subacute phase. The authors demonstrated that the device not only improved WM but also cognition and psychological health (14), as well as activity of daily living, as reported also by Johansson & Tornmalm (15). According to these results, Lundqvist et al. (16) performed a cross-over design controlled experimental study using CogMed software on 21 subjects with ABI. They observed significant improvement in WM tasks, occupational performance, performance satisfaction, and overall health rating (16). Svaerke et al. found similar results in a randomized study of 72 patients (17). Moreover, these findings could be generalized to the life context, as suggested by Johansson et al. (15). Improvements have been observed also in other neurological populations, including multiple sclerosis (MS) (18). A study on older adults with Mild Cognitive Impairment (MCI) showed an improvement in cognitive skills, especially in information processing speed and WM, after specific home interventions (51).

On the other hand, Nyberg et al. (19) conducted a study in 26 stroke patients trained with CogMed for 6 weeks. The authors found changes in performance related to the trained computerized task, but no microstructural changes in white matter between rest and training condition (*p* = 0.99).

### 3.2. Lumosity™

Lumosity™ (*Brain Games Lumos Labs. Lumosity: Recover Your Brain™. San Francisco, Calif.: Dakim, Inc.; 2010*) is a CR software that provides access to games to improve cognitive processing speed, flexibility, attention, memory, and problem-solving skills. However, the results of its effectiveness are conflicting.

Withiel et al. found a good usability of the device, especially for its playful aspects, but without improvements in daily memory, in 20 stroke survivors (20). These results were confirmed by Wentink et al. that carried out an experimental study on chronic stroke patients. The authors showed no effect of training on cognitive functioning, QoL, or self-efficacy regarding the control condition, except for minimal effects on WM and speed (21).

Conversely, Stuifbergen et al. performed a study on 183 MS patients, noting that the device is feasible with promising effects in improving cognitive functioning (22). Furthermore, Zickefoose et al. evaluated whether the treatment of severe traumatic brain injury (TBI) survivors could be generalized to comparable, untrained tasks. They found that participants made significant improvements but with limited generalization (23). Finally, evidence is inconsistent regarding the effectiveness of this device on subjective or objective memory or other cognitive components.

### 3.3. BrainHQ

BrainHQ (*Posit Science Corporation, San Francisco CA, 2015*) is an online cognitive training system for cognitive exercises. Each user can be monitored throughout the entire training, which is automatically modified according to the skill level reached. BrainHQ has multiple exercises for training different cognitive skills, including attention, speed, memory, sociability, orientation, and intelligence. The program appears to have good feasibility and effective results in CR.

Yeh et al. performed a randomized controlled trial to evaluate the efficacy of a combination of aerobic exercise and cognitive training using BrainHQ on stroke survivors. The authors noticed that, compared to the control group, the experimental group significantly improved global cognitive functioning and memory scores after training (24). These results were confirmed by Charvet et al. (25) in patients with MS, demonstrating how home computer-based cognitive training can improve cognitive functioning. Furthermore, they observed that this telerehabilitation approach enabled good patient compliance and rapid recruitment (25). Finally, an interesting pilot study by O’Neil-Pirozzi et al. explored the feasibility and effects of participating in a computerized cognitive fitness exercise program on ABI adults with positive results (26).

### 3.4. Neuro PersonalTrainer^®^

Neuro PersonalTrainer®-MH (*GNPT®, Guttmann Institute, Badalona, Spain, 2011*) is a module for neurocognitive rehabilitation provided by a computerized tele-rehabilitation platform. It allows one to carry out cognitive training in an intensive and personalized mode (27). Gil-Pages et al. in their cross-over, randomized, controlled, double-blind clinical study observed that chronic stroke patients with cognitive impairment may benefit from cognitive training using this innovative tool (27). On the contrary, Aparicio-López et al., in a randomized clinical trial of 28 stroke patients, found no statistically significant differences when comparing patients using the Neuropersonal-Trainer to those receiving traditional pc-based rehabilitation (52).

### 3.5. ERICA

ERICA (*Giunti Psychometrics, Italy, 2013*) is a tool composed of a series of computerized exercises for cognitive rehabilitation. These exercises are dedicated to the rehabilitation of specific skills, such as attention, spatial cognition, memory, verbal executive functions, and non-verbal executive functions, and can be used in patients with neuropsychological deficits resulting from brain injury, developmental disorders, degenerative pathologies, and psychiatric pathologies. The studies using this device mainly involve subjects suffering from Parkinson’s disease (PD), and multiple sclerosis (MS). DeLuca et al. (28) performed a randomized clinical study on 70 PD patients, noting significant improvement after CR in both groups. However, the group receiving the Erica training achieved greater outcomes, especially in attention, orientation, and visuospatial domains (28). The same research group observed similar significant improvements in people with MS (29). The positive effects of Erica on the emotional, motor, and cognitive aspects in MS patients were also highlighted by Barburulo et al. in a study of 63 MS patients (30).

### 3.6. CogniPlus

CogniPlus (*Schuhfried GmbH, Vienna, Austria, 2008*) is a tool related to the Vienna Test System, which integrates the diagnosis, treatment, and assessment of various cognitive functions, such as attention, executive functions, memory, spatial processing, and visuomotor abilities. Cogniplus has been shown to be effective in CR. Hagovská et al. (31) performed a study to compare the effectiveness of two types of cognitive training in 60 older adults with MCI. The results showed that although both traditional and experimental groups had an improvement, the Cogniplus group reported better scores in quality of life and better attention (31).

Cogniplus is also effective in combination treatments. Westerhof-Evers et al. (53) conducted a study to evaluate the effects of treatment using Cogniplus combined with T-scEmo (a tool that affects emotions) on social cognition and emotion regulation in 61 TBI patients. The authors noticed that this combined approach may be effective in rehabilitating impairments in social cognition (53). Another study by Hagovská et al. (54) on 80 elderly participants with MCI showed that Cogniplus can improve balance control, cognitive functions, gait speed, and activities of daily living, when combined to motor interventions (54).

In contrast to these studies, Zimmerman et al. (32) performed a study on patients with Parkinson’s disease (PD) using cognitive training with Cogniplus and motor training with a movement game in different groups. They found that specific computer training for cognition is not superior to a motion-controlled computer game in improving cognitive performance (32).

### 3.7. Attention process training

APT (*Lash & Associates Publishing/Training Inc, Youngsville, North Carolina, 2010*) is a clinical program used for attention process training in adolescents, adults, and older adults with ABI. It was developed by Sohlberg & Mateer, and it is based on scientific evidence, as it has demonstrated its effectiveness in the rehabilitation of patients with cognitive disorders (55).

Pantoni et al. (33) carried out a single-blind randomized clinical trial to evaluate the effects of CR in 46 patients with MCI, using the Attention Process Training (APT) program. The authors found that APT potentially enhances focused attention and WM and appears to increase activity in brain circuits involved in cognition (33). APT training also seems to be effective in other patient populations. Walton et al. (34) carried out a randomized study of 65 PD patients to evaluate whether targeted training could improve freezing and executive dysfunction. The results highlighted that APT training can be an effective method to improve processing speed and reduce daytime sleepiness (34).

### 3.8. CoTras

The CoTras program (*RPIO Co., Ltd., Geumcheon-gu, Seoul, 2010*) is a computer-based cognitive rehabilitation device. It consists of real-life training content which is defined according to the environment in Korea. It has several exercises that adapt to the patient’s cognitive abilities, including difficulty, time, and speed of exercise execution. Park and Park (35) carried out a study to investigate the effects of CoTras on cognition in thirty acute stroke patients. The results showed that the tool can stimulate the recovery of global cognitive function, with regard to and visual perception (35).

### 3.9. BrainGymmer

BrainGymmer (*Dezzel Media, The Netherlands, 2010*) consists of computer-based cognitive training exercises via a website. The training tasks consist of games designed to be challenging and customized to the characteristics of the user.

Van de Ven et al. (36) carried out a double-blind, randomized controlled trial to investigate whether the computer-based training improves executive functioning after stroke. The results showed that patients submitted to Braingymmer training had the same improvement in executive and general cognitive functioning as control groups. This improvement was likely due to non-specific training effects. Therefore, the Braingymmer program does not seem to make significantly different improvements compared to conventional methods. Nevertheless, other studies on larger samples should be implemented to ascertain the effectiveness of this tool.

### 3.10. RehaCom^®^

RehaCom (*HASOMED GmbH, Magdeburg, Germany, 1997*) is a software for computer-assisted cognitive rehabilitation useful in the management of different cognitive disorders. The system supports recovery and replacement processes, potentiating cognitive strategies and offering targeted therapeutic solutions for rehabilitation.

Various studies have shown positive results of intervention using Rehacom, even in telerehabilitation modality, to improve or stabilize cognitive decline. Nousia et al. (37) carried out a study on 46 Greek patients with MCI. The authors demonstrated the efficacy of Rehacom on delayed and semantic memory, word recognition, and attentional shifting. The results have been confirmed by other authors. Naeeni Davarani et al. (38) investigated the effect of RehaCom on attention, response control, processing speed, working memory, visuospatial skills, and verbal/nonverbal executive functions in 60 MS patients. They observed that RehaCom treatment improved all cognitive functions, and this effect was maintained over time (i.e., at three-month follow-up) (38). Moreover, Amir et al. (39) carried out a study of 50 stroke survivors. They showed a significant improvement in working memory and processing speed in the experimental group compared to the control group after a 5-week training with the software (39). These results were confirmed by Messinis et al. (40), who carried out a randomized controlled study to examine the efficacy of at-home intervention using RehaCom software in 36 patients with secondary progressive MS. The authors found that the tool can be effective in improving cognitive functioning and mood with positive results on fatigue and health-related quality of life (40). These findings were confirmed by a multicenter study carried out by the same authors (41) on 58 MS patients. In fact, the authors showed significant improvements in episodic memory, information processing speed/attention, and executive functions with a positive perception of patients in using the training software RehaCom (41). Moreover, Campbell et al. (42) explored the efficacy of home-based computer-aided cognitive rehabilitation in 38 patients with MS using neuropsychological assessment and advanced structural and functional MRI. The treatment group had greater activation in the bilateral prefrontal cortex and right temporoparietal regions. In addition, improved cognitive performance was noted in patients treated with Rehacom (42). Finally, Bonavita et al. (43) performed a study on 18 relapsing–remitting MS patients treated with Rehacom software. They demonstrated that training with the software can induce an adaptive cortical reorganization as well as better cognitive performance (43).

Darestani et al. (44) conducted research to investigate the effect of RehaCom treatment on verbal performance in 60 MS patients. The results showed that treatment with the software can improve speech fluency, verbal learning, and memory in MS patients (44).

Veisi-Pirkoohi et al. (45) found that RehaCom rehabilitation software was effective on ADL, attention, and response control in 50 chronic stroke patients due to middle and anterior cerebral arteries occlusion (45). Yoo et al. (7), in their study on 46 patients with stroke, found that computer-assisted cognitive rehabilitation with the RehaCom program improved cognitive function. This raises the idea that the tool may be helpful for stroke patients who have cognitive impairment (7). Fernández et al. (46) investigated the effectiveness of the software on patients with ABI. The authors showed a good efficacy of the training procedure in focused attention, digit span, and logical and working memory (46). In another study performed on 50 hospitalized patients (56), the same authors found an improvement in the trained functions in all patients. However, adverse effects, including mental fatigue, headaches, and eye irritation, have been found to negatively affect the usability of the tool (56).

Finally, an interesting randomized controlled trial (47) was carried out on 8 patients with PD. The authors found that the patients improved attention and processing speed with changes in neural plasticity, as investigated by fMRI (47).

### 3.11. GRADIOR

GRADIOR (INTRAS Foundation, Spain) is a multimedia software for cognitive stimulation, neuropsychological assessment, and rehabilitation. It consists of personalized exercises that train various cognitive domains, such as attention, memory, orientation, calculation, perception, reasoning, and language. This software creates a multimedia environment with high flexibility and demanding challenges that boost the cognitive components. The use of the software requires the presence of a qualified therapist to support the user during the assessment and training. Few studies have evaluated its usability and effectiveness in the rehabilitation field. Diaz Baquero et al. performed an RCT study on 43 patients with MCI and mild dementia, highlighting good adherence to treatment, good acceptability, and potential efficacy of the device (8). Another RCT performed by the same authors on 89 people with MCI and dementia demonstrated the benefit of this training on several cognitive domains (48). These promising results were confirmed by Gongora Alonso et al., who observed good acceptability of the tool in patients with severe and prolonged mental illness (49). Finally, Vanova et al. performed an RCT of 400 people with MCI and mild dementia treated with Gradior. They found significant improvements in most patients, with long-term maintenance of the results (50).

## 4. Discussion

This review aimed to identify suitable technological devices for the CR of chronic neurological patients. Specifically, our literature research has shown how these devices can be used with different neurological pathologies, including stroke, MS, TBI and PD. In detail, it emerges that the clinical population with the most trials is stroke (*N* = 10) (19–21, 24, 29, 35, 36, 39, 45, 52), followed by MS (*N* = 9) (18, 22, 25, 30, 38, 40–43), PD (*N* = 4) (28, 32, 34, 47), and traumatic and acquired brain injury (*N* = 4, respectively) (14, 17, 23, 26). Only two studies investigating patients with different neurological pathologies were recruited (15, 16), as reported in Table 2. The misrepresentation of RCT studies with such a different neurological population could be intrinsically linked to the difficulty in managing patients affected, e.g., by TBI and dementia. For dementia, there is some evidence that computer-based cognitive rehabilitation may be of help in improving different cognitive domains (57). In particular, the software “GRADIOR” looks promising in the CR field (8, 48–50). Moreover, previous studies have applied computerized approaches using photos of the patient and his/her personal surroundings, with positive results (58, 59). However, these studies were excluded for temporal reasons.

Moreover, most studies reported a statistically significant efficacy of using the PC-based devices in reparative CR. However, only in some cases they were superior to conventional treatments. Training duration, frequency and timing is still unclear. For CogMed, 5 weeks of intervention with each session lasting between 30 and 45 min seems to be the best solution for different populations of patients. However, the efficacy was mainly observed within the group, and not with respect to the control group (14–19). Luminosity™ was mainly used in patients with stroke, but also with MS and TBI, with an intervention duration ranging from 4 to 12 weeks (each session lasting from 20 to 45 min) and with an efficacy higher than that observed for the control group (20–23). Brain HQ needed longer intervention times (12–20 weeks, each session lasting 30–60 min), but with a higher efficacy reported with respect to control intervention for patients with stroke, MS and TBI (24–26). Three studies investigated the use of ERICA for 8–24 weeks (each session 45–60 min), demonstrating significant results only within the experimental group (28–30). CogniPlus (31, 32) and APT (33, 34) have been used for patients with MCI or PD with high variability in the duration of interventions. CoTras (35) and BrainGymmer (36) were both tested in a single study on patients with stroke, the former for a shorter period (4 vs. 12 weeks) and with between group significant differences.

RehaCom was the device more widely tested, especially in patients with MS. The high number of studies increased the variability of the adopted protocols, with a duration of the intervention going from 5 to 15 weeks (each session ranged between 30 and 60 min). However, literature on this device reports solid statistically significant results about its efficacy also when compared to conventional interventions (7, 37–47).

Therefore, we noticed that the importance of training cognitive functions is increasingly evident in the literature, also for facilitating learning processes in motor recovery (4, 60). Indeed, computerized cognitive rehabilitation has proven effective in combination with other methods. A practical example can be the application of acupuncture coupled to transcranial direct current stimulation with computerized cognitive rehabilitation. This method showed good results in cognitive performance in individuals with vascular cognitive impairment (61) and people with stroke (62). A recent study by Shaker et al. (62) demonstrated significant improvement in scores of attention and concentration domains, figural memory, logical reasoning, and reaction times performance. People with cognitive disabilities are treated intensively in the subacute stage of the disease, while unfortunately, they have little access to treatment in the chronic stage. This problem is due to the burden of the Local Health Care Institutions. The underestimation as well as the reduced possibility of effective cognitive training after subacute rehabilitation regards both subjects with central nervous system pathology and those affected by other conditions, such as for example non-CNS cancer (63). This is why new solutions, including telemedicine and home devices/software for cognitive rehabilitation, may be helpful to guarantee the continuity of care. In fact, they should be used when geographical and socio-economic barriers prevent the patient from reaching primary clinics. This will allow each patient to receive monitoring and rehabilitation, through remote devices (3, 64–66).

Moreover, the patient’s perception of the device usability is a key point of rehabilitation. In fact, recent studies have pointed out that the adaptability of technology also includes adapting to patients’ emotions or perceptions. An interesting study by Norman et al. (65) pointed out that perception of a device influences the use of that device itself (37). Nonetheless, this aspect deserves further investigation, as some tools could have high costs and reduce the possibility of customizing the design of the tools. Although in the last period very flexible low-cost proposals have been advanced, also based on smartphones and apps to download for free. We have not explored this field as they are out of the scope of this review. Possible problems concerning the diffusion and use of such devices at home could concern: (i) the absence of a caregiver to supervise the training, especially for patients with greater impairment and with a greater need for therapy; (ii) the lack of experience with technological interfaces and PCs by both patient and caregiver; (iii) the lack of structural technical requirements such as not having a PC or an internet connection. Similar issues have recently been raised by Mantovani et al. (67) concerning the use of Virtual Reality as a home therapy for CR.

In general, it seems that the use of technological devices for CR is promising, but with inconsistencies due to the variations in study design. However, we must bracket the proposal with a caveat. Although these technological devices have features that make them highly adaptive to the patient’s performance. For more severe and subacute subjects they cannot replace conventional CR, in which neuropsychologists and speech therapists play a fundamental role. In fact, their optimal use always remains integrated with conventional CR, or they are part of a rehabilitation process following discharge, to support the patient remotely. However, the protocols of the various studies are very different both in the frequency and the duration of the sessions. This makes it difficult to judge the effectiveness of the tool, so new randomized trials with large samples should be conducted to confirm this aspect. Moreover, it is important that clinicians are familiar with the different devices, in order to facilitate the selection of the appropriate device for the treatment to be performed. Finally, another problem is related to the difficulty of standardizing tests for patients with different neurological pathologies. This implies the need to validate tests for different patients, favoring the continuous updating of devices and tests. Indeed, young subjects, such as those with MS, are more familiar with computerized devices and may require different tests than patients with MCI. Often young patients stop testing because they get bored, or quickly reach the various levels of the tests. On the other hand, patients with dementia and severe cognitive decline may have serious difficulties in using the devices. This could be the main reason why we did not find studies in patients with dementia.

Our review had the ambitious aim of offering an overview of the devices currently in use in clinical practice for the computerized CR of neurological patients. We have collected many studies with the aim of describing the devices and highlighting their strengths and weaknesses. A wide variability among the revised papers was noted in terms of primary as well as secondary outcome measures, even when aiming at measuring the same cognitive domain. This is accompanied by a wide variability also in the duration of treatments, including both session duration and length of rehabilitative period in which a specific device was used (as shown in Table 2). There is the need to standardize assessment and rehabilitative protocols by identifying the key parameter for each device. The inter-rater reliability in the coding and interpretation of these parameters, which in this review cannot be performed given the wide variability among the studies. Thus, this work has limitations. Unlike validation studies, it is not possible to operationalize and define the key parameters being analyzed in the identified literature and then demonstrate inter-rater reliability in the coding or interpretation of each of the defined parameters. The scientific literature on this topic is very varied: different devices are used, for different types of patients, administering a different amount of therapies/duration. Further meta-analysis reviews are needed to fulfill this purpose. In the near future, various factors can consolidate and improve the possibility of carrying out cognitive therapy using software and platforms at home. They include: (i) better accessibility (in terms of lower costs and greater geographical coverage), (ii) higher attention to the chronic and territorial phase of neurorehabilitation and (iii) a growing sensitivity to the possibility of ensuring a better quality of life for brain injury survivors.

With this in mind and considering the aforementioned limitations, PC based approaches could be valuable complementary tools to improve cognitive function and partly guarantee the continuity of care in neurological patients.

## Author contributions

MM: Conceptualization, Formal analysis, Methodology, Writing – original draft. DB: Formal analysis, Investigation, Methodology, Writing – original draft. RC: Investigation, Methodology, Writing – original draft, Writing – review & editing. IC: Supervision, Validation, Writing – review & editing. AC: Validation, Writing – review & editing, Supervision. PT: Supervision, Visualization, Writing – review & editing. FI: Software, Validation, Writing – review & editing. SP: Software, Writing – review & editing. GA: Software, Validation, Writing – review & editing. GM: Methodology, Visualization, Writing – review & editing. MI: Supervision, Visualization, Writing – review & editing.

## References

[1] Md Fadzil NH Shahar S Rajikan R Singh DKA Mat Ludin AF Subramaniam P. A scoping review for usage of Telerehabilitation among older adults with mild cognitive impairment or cognitive frailty. Int J Environ Res Public Health. (2022) 19:4000. doi: 10.3390/ijerph19074000, PMID: 35409683 PMC8997970

[2] Xiang YT Zhao YJ Liu ZH Li XH Zhao N Cheung T. The COVID-19 outbreak and psychiatric hospitals in China: managing challenges through mental health service reform. Int J Bio Sci. (2020) 16:1741–4. doi: 10.7150/ijbs.45072, PMID: 32226293 PMC7098035

[3] Maggio MG De Luca R Manuli A Calabrò RS. The five 'W' of cognitive telerehabilitation in the COVID-19 era. Expert Rev Med Devices. (2020) 17:473–5. doi: 10.1080/17434440.2020.1776607, PMID: 32476504

[4] Morone G Spitoni GF De Bartolo D. Rehabilitative devices for a top-down approach. Expert Rev Med Devices. (2019) 16:187–95. doi: 10.1080/17434440.2019.1574567, PMID: 30677307

[5] Vilou I Bakirtzis C Artemiadis A Ioannidis P Papadimitriou M Konstantinopoulou E. Computerized cognitive rehabilitation for treatment of cognitive impairment in multiple sclerosis: an explorative study. J Integr Neurosci. (2020) 19:341–7. doi: 10.31083/j.jin.2020.02.35, PMID: 32706198

[6] Sampanis DS Mevorach C Shalev L. Cognitive deficits after stroke through computerized progressive attentional training (CPAT): a pilot study. Phys Med Rehabil Int. (2015) 2:1058.

[7] Yoo C Yong MH Chung J Yang Y. Effect of computerized cognitive rehabilitation program on cognitive function and activities of living in stroke patients. J Phys Ther Sci. (2015) 27:2487–9. doi: 10.1589/jpts.27.2487, PMID: 26355244 PMC4563296

[8] Diaz Baquero AA Perea Bartolomé MV Toribio-Guzmán JM Martínez-Abad F Parra Vidales E Bueno Aguado Y. Determinants of adherence to a "GRADIOR" computer-based cognitive training program in people with mild cognitive impairment (MCI) and mild dementia. J Clin Med. (2022) 11:1714. doi: 10.3390/jcm11061714, PMID: 35330040 PMC8955227

[9] Zhou X Snoswell CL Harding LE Bambling M Edirippulige S Bai X. The role of telehealth in reducing the mental health burden from COVID-19. Telemed J E Health. (2020) 26:377–9. doi: 10.1089/tmj.2020.0068, PMID: 32202977

[10] De Cola MC De Luca R Bramanti A Bertè F Bramanti P Calabrò RS. Tele-health services for the elderly: a novel southern Italy family needs-oriented model. J Telem Telecare. (2016) 22:356–62. doi: 10.1177/1357633X15604290, PMID: 26377125

[11] Burns SP Terblanche M Perea J. mHealth intervention applications for adults living with the effects of stroke: a scoping review. Arch Rehabil Res Clin Transl. (2020) 3:100095. doi: 10.1016/j.arrct.2020.100095 33778470 PMC7984984

[12] Meulenberg CJW de Bruin ED Marusic U. A perspective on implementation of technology-driven Exergames for adults as Telerehabilitation services. Front Psychol. (2022) 13:840863. doi: 10.3389/fpsyg.2022.840863, PMID: 35369192 PMC8968106

[13] Bernini S Panzarasa S Sinforiani E Quaglini S Cappa SF Cerami C. HomeCoRe for Telerehabilitation in mild or major neurocognitive disorders: a study protocol for a randomized controlled trial. Front Neurol. (2021) 12:752830. doi: 10.3389/fneur.2021.752830, PMID: 35002919 PMC8733654

[14] Akerlund E Esbjörnsson E Sunnerhagen KS Björkdahl A. Can computerized working memory training improve impaired working memory, cognition and psychological health? Brain Inj. (2013) 27:1649–57. doi: 10.3109/02699052.2013.830195, PMID: 24087909

[15] Johansson B Tornmalm M. Working memory training for patients with acquired brain injury: effects in daily life. Scand J Occup Ther. (2012) 19:176–83. doi: 10.3109/11038128.2011.603352, PMID: 21843045

[16] Lundqvist A Grundström K Samuelsson K Rönnberg J. Computerized training of working memory in a group of patients suffering from acquired brain injury. Brain Inj. (2010) 24:1173–83. doi: 10.3109/02699052.2010.498007, PMID: 20715888

[17] Svaerke K Pyke SB Tjoernlund M Humle F Mogensen J. Effects of computer-based cognitive rehabilitation on working memory in patients with acquired brain injury in the chronic phase, a pilot-study. Brain Inj. (2022):1–11. doi: 10.1080/02699052.2022.2034965 35157537

[18] Blair M Goveas D Safi A Marshall C Rosehart H Orenczuk S. Does cognitive training improve attention/working memory in persons with MS? A pilot study using the Cogmed working memory training program. Mult Scler Relat Disord. (2021) 49:102770. doi: 10.1016/j.msard.2021.102770, PMID: 33497850

[19] Nyberg CK Nordvik JE Becker F Rohani DA Sederevicius D Fjell AM. A longitudinal study of computerized cognitive training in stroke patients – effects on cognitive function and white matter. Top Stroke Rehabil. (2018) 25:241–7. doi: 10.1080/10749357.2018.1443570, PMID: 29480129

[20] Withiel TD Wong D Ponsford JL Cadilhac DA New P Mihaljcic T. Comparing memory group training and computerized cognitive training for improving memory function following stroke: a phase II randomized controlled trial. J Rehabil Med. (2019) 51:343–51. doi: 10.2340/16501977-2540, PMID: 30815708

[21] Wentink MM Berger MA de Kloet AJ Meesters J Band GP Wolterbeek R. The effects of an 8-week computer-based brain training programme on cognitive functioning, QoL and self-efficacy after stroke. Neuropsychol Rehabil. (2016) 26:847–65. doi: 10.1080/09602011.2016.1162175, PMID: 27184585

[22] Stuifbergen AK Becker H Perez F Morrison J Brown A Kullberg V. Computer-assisted cognitive rehabilitation in persons with multiple sclerosis: results of a multi-site randomized controlled trial with six month follow-up. Disabil Health J. (2018) 11:427–34. doi: 10.1016/j.dhjo.2018.02.001, PMID: 29477372 PMC6047944

[23] Zickefoose S Hux K Brown J Wulf K. Let the games begin: a preliminary study using attention process training-3 and Lumosity™ brain games to remediate attention deficits following traumatic brain injury. Brain Inj. (2013) 27:707–16. doi: 10.3109/02699052.2013.775484, PMID: 23672446

[24] Yeh TT Chang KC Wu CY. The active ingredient of cognitive restoration: a multicenter randomized controlled trial of sequential combination of aerobic exercise and computer-based cognitive training in stroke survivors with cognitive decline. Arch Phys Med Rehabil. (2019) 100:821–7. doi: 10.1016/j.apmr.2018.12.020, PMID: 30639273

[25] Charvet LE Yang J Shaw MT Sherman K Haider L Xu J. Cognitive function in multiple sclerosis improves with telerehabilitation: results from a randomized controlled trial. PLoS One. (2017) 12:e0177177. doi: 10.1371/journal.pone.0177177, PMID: 28493924 PMC5426671

[26] O'Neil-Pirozzi TM Hsu H. Feasibility and benefits of computerized cognitive exercise to adults with chronic moderate-to-severe cognitive impairments following an acquired brain injury: a pilot study. Brain Inj. (2016) 30:1617–25. doi: 10.1080/02699052.2016.1199906, PMID: 27680422

[27] Gil-Pagés M Solana J Sánchez-Carrión R Tormos JM Enseñat-Cantallops A García-Molina A. A customized home-based computerized cognitive rehabilitation platform for patients with chronic-stage stroke: study protocol for a randomized controlled trial. Trials. (2018) 19:191. doi: 10.1186/s13063-018-2577-8, PMID: 29566766 PMC5863836

[28] De Luca R Latella D Maggio MG. Computer assisted cognitive rehabilitation improves visuospatial and executive functions in Parkinson's disease: preliminary results. NeuroRehabilitation. (2019) 45:285–90. doi: 10.3233/NRE-192789, PMID: 31498141

[29] De Luca R Leonardi S Spadaro L. Improving cognitive function in patients with stroke: can computerized training be the future? J Stroke Cerebrovasc Dis. (2018) 27:1055–60. doi: 10.1016/j.jstrokecerebrovasdis.2017.11.008, PMID: 29221967

[30] Barbarulo AM Lus G Signoriello E Trojano L Grossi D Esposito M. Integrated cognitive and Neuromotor rehabilitation in multiple sclerosis: a pragmatic study. Front Behav Neurosci. (2018) 12:196. doi: 10.3389/fnbeh.2018.00196, PMID: 30271331 PMC6146227

[31] Hagovská M Dzvoník O Olekszyová Z. Comparison of two cognitive training programs with effects on functional activities and quality of life. Res Gerontol Nurs. (2017) 10:172–80. doi: 10.3928/19404921-20170524-01, PMID: 28556876

[32] Zimmermann R Gschwandtner U Benz N Hatz F Schindler C Taub E. Cognitive training in Parkinson disease: cognition-specific vs nonspecific computer training. Neurology. (2014) 82:1219–26. doi: 10.1212/WNL.0000000000000287, PMID: 24623840

[33] Pantoni L Poggesi A Diciotti S Valenti R Orsolini S Della Rocca E. Effect of attention training in mild cognitive impairment patients with subcortical vascular changes: the RehAtt study. J Alzheimers Dis. (2017) 60:615–24. doi: 10.3233/JAD-170428, PMID: 28869475 PMC5611829

[34] Walton CC Mowszowski L Gilat M Hall JM O’Callaghan C Muller AJ. Cognitive training for freezing of gait in Parkinson's disease: a randomized controlled trial. NPJ Parkinsons Dis. (2018) 4:15. doi: 10.1038/s41531-018-0052-6, PMID: 29796409 PMC5959878

[35] Park JH Park JH. The effects of a Korean computer-based cognitive rehabilitation program on cognitive function and visual perception ability of patients with acute stroke. J Phys Ther Sci. (2015) 27:2577–9. doi: 10.1589/jpts.27.2577, PMID: 26356152 PMC4563318

[36] van de Ven RM Buitenweg JI Schmand B Veltman DJ Aaronson JA Nijboer TC. Brain training improves recovery after stroke but waiting list improves equally: a multicenter randomized controlled trial of a computer-based cognitive flexibility training. PLoS One. (2017) 12:e0172993. doi: 10.1371/journal.pone.0172993, PMID: 28257436 PMC5336244

[37] Nousia A Martzoukou M Siokas V Aretouli E Aloizou AM Folia V. Beneficial effect of computer-based multidomain cognitive training in patients with mild cognitive impairment. Appl Neuropsych Adult. (2021) 28:717–26. doi: 10.1080/23279095.2019.1692842, PMID: 31885287

[38] Naeeni Davarani M Arian Darestani A Hassani-Abharian P Vaseghi S Zarrindast MR Nasehi M. RehaCom rehabilitation training improves a wide-range of cognitive functions in multiple sclerosis patients. Appl Neuropsychol Adult. (2022) 29:262–72. doi: 10.1080/23279095.2020.1747070, PMID: 32368936

[39] Amiri S Hassani-Abharian P Vaseghi S Kazemi R Nasehi M. Effect of RehaCom cognitive rehabilitation software on working memory and processing speed in chronic ischemic stroke patients. Assist Technol. (2021) 35:41–7. doi: 10.1080/10400435.2021.1934608 34033513

[40] Messinis L Kosmidis MH Nasios G Konitsiotis S Ntoskou A Bakirtzis C. Do secondary progressive multiple sclerosis patients benefit from computer- based cognitive neurorehabilitation? A randomized sham controlled trial. Mult Scler Relat Disord. (2020) 39:101932. doi: 10.1016/j.msard.2020.101932, PMID: 31927200

[41] Messinis L Nasios G Kosmidis MH Zampakis P Malefaki S Ntoskou K. Efficacy of a computer-assisted cognitive rehabilitation intervention in relapsing-remitting multiple sclerosis patients: a multicenter randomized controlled trial. Behav Neurol. (2017) 2017:1–17. doi: 10.1155/2017/5919841 PMC5804109 29463950

[42] Campbell J Langdon D Cercignani M Rashid W. A randomized controlled trial of the efficacy of cognitive rehabilitation in multiple sclerosis: a cognitive, behavioral, and MRI study. Neural Plastic. (2016) 2016:1–9. doi: 10.1155/2016/4292585 PMC5223046 28116167

[43] Bonavita S Sacco R Della Corte M Esposito S Sparaco M d’Ambrosio A. Computer-aided cognitive rehabilitation improves cognitive performances and induces brain functional connectivity changes in relapsing remitting multiple sclerosis patients: an exploratory study. J Neurol. (2015) 262:91–100. doi: 10.1007/s00415-014-7528-z, PMID: 25308631

[44] Arian Darestani A Naeeni Davarani M Hassani-Abharian P Zarrindast MR Nasehi M. The therapeutic effect of treatment with RehaCom software on verbal performance in patients with multiple sclerosis. J Clin Neurosci. (2020) 72:93–7. doi: 10.1016/j.jocn.2020.01.007, PMID: 31937503

[45] Veisi-Pirkoohi S Hassani-Abharian P Kazemi R Vaseghi S Zarrindast MR Nasehi M. Efficacy of cognitive rehabilitation software RehaCom in activities of daily living, attention and response control in chronic stroke patients. J Clin Neurosci. (2020) 71:101–7. doi: 10.1016/j.jocn.2019.08.114, PMID: 31495655

[46] Fernandez E Bergado Rosado JA Rodriguez Perez D Salazar Santana S Torres Aguilar M Bringas ML. Effectiveness of a computer-based training program of attention and memory in patients with acquired brain damage. Behav Sci (Basel). (2017) 8:4. doi: 10.3390/bs8010004, PMID: 29301194 PMC5791022

[47] Cerasa A Gioia MC Salsone M Donzuso G Chiriaco C Realmuto S. Neurofunctional correlates of attention rehabilitation in Parkinson's disease: an explorative study. Neurol Sci. (2014) 35:1173–80. doi: 10.1007/s10072-014-1666-z, PMID: 24554416

[48] Diaz Baquero AA Franco-Martín MA Parra Vidales E Toribio-Guzmán JM Bueno-Aguado Y Martínez Abad F. The effectiveness of GRADIOR: a neuropsychological rehabilitation program for people with mild cognitive impairment and mild dementia. Results of a randomized controlled trial after 4 and 12 months of treatment. J Alzheimers Dis. (2022) 86:711–27. doi: 10.3233/JAD-215350, PMID: 35124649 PMC9028667

[49] Góngora Alonso S Fumero Vargas G Morón Nozaleda L Sainz de Abajo B de la Torre DI Franco M. Usability analysis of a system for cognitive rehabilitation, "Gradior", in a Spanish region. Telemed J E Health. (2020) 26:671–82. doi: 10.1089/tmj.2019.0084 31545150

[50] Vanova M Irazoki E García-Casal JA Martínez-Abad F Botella C Shiells KR. The effectiveness of ICT-based neurocognitive and psychosocial rehabilitation programmes in people with mild dementia and mild cognitive impairment using GRADIOR and ehcoBUTLER: study protocol for a randomised controlled trial. Trials. (2018) 19:100. doi: 10.1186/s13063-017-2371-z, PMID: 29433545 PMC5810083

[51] Wayne RV Hamilton C Jones Huyck J Johnsrude IS. Working memory training and speech in noise comprehension in older adults. Front Aging Neurosci. (2016) 8:49. doi: 10.3389/fnagi.2016.00049 27047370 PMC4801856

[52] Aparicio-López C García-Molina A García-Fernández J López-Blázquez R Enseñat-Cantallops A Sánchez-Carrión R. Combination treatment in the rehabilitation of visuo-spatial neglect. Psicothema. (2016) 28:143–9. doi: 10.7334/psicothema2015.93, PMID: 27112810

[53] Westerhof-Evers HJ Visser-Keizer AC Fasotti L Schönherr MC Vink M van der Naalt J. Effectiveness of a treatment for impairments in social cognition and emotion regulation (T-ScEmo) after traumatic brain injury: a randomized controlled trial. J Head Trauma Rehabil. (2017) 32:296–307. doi: 10.1097/HTR.0000000000000332, PMID: 28786854

[54] Hagovská M Olekszyová Z. Relationships between balance control and cognitive functions, gait speed, and activities of daily living. Z Gerontol Geriatr. (2016) 49:379–85. doi: 10.1007/s00391-015-0955-3, PMID: 26458911

[55] Sohlberg MM Mateer CA. Effectiveness of an attention-training program. J Clin Exp Neuropsychol. (1987) 9:117–30. doi: 10.1080/01688638708405352 3558744

[56] Fernández E Bringas ML Salazar S Rodríguez D García ME Torres M. Clinical impact of RehaCom software for cognitive rehabilitation of patients with acquired brain injury. MEDICC Rev. (2012) 14:32–5. doi: 10.37757/MR2012V14.N4.8, PMID: 23154316

[57] Klimova B Maresova P. Computer-based training programs for older people with mild cognitive impairment and/or dementia. Front Hum Neurosci. (2017) 11:262. doi: 10.3389/fnhum.2017.00262, PMID: 28559806 PMC5432561

[58] Hofmann M Hock C Kühler A Müller-Spahn F. Interactive computer-based cognitive training in patients with Alzheimer's disease. J Psychiatr Res. (1996) 30:493–501. doi: 10.1016/S0022-3956(96)00036-2, PMID: 9023793

[59] Hofmann M Hock C Müller-Spahn F. Computer-based cognitive training in Alzheimer's disease patients. Ann N Y Acad Sci. (1996) 777:249–54. doi: 10.1111/j.1749-6632.1996.tb34427.x, PMID: 8624093

[60] Park HK Song MK Kim JH Han JY. A randomized controlled trial to evaluate the effectiveness and safety of electro acupuncture and transcranial direct current stimulation with computerized cognitive rehabilitation in patients with vascular cognitive impairment. Medicine (Baltimore). (2020) 99:e21263. doi: 10.1097/MD.0000000000021263, PMID: 32702911 PMC7373529

[61] Jiang C Yang S Tao J Huang J Li Y Ye H. Clinical efficacy of acupuncture treatment in combination with RehaCom cognitive training to improve cognitive function in stroke: a factorial 2 × 2 design a randomized controlled trial. J Am Med Dir Assoc. (2016) 17:1114–22. doi: 10.1016/j.jamda.2016.07.021, PMID: 27592180

[62] Shaker HA Sawan SAE Fahmy EM Ismail RS Elrahman SAEA. Effect of transcranial direct current stimulation on cognitive function in stroke patients. Egypt J Neurol Psychiatr Neurosurg. (2018) 54:32. doi: 10.1186/s41983-018-0037-8, PMID: 30459505 PMC6223736

[63] De Luca R Torrisi M Bramanti A. A multidisciplinary telehealth approach for community dwelling older adults. Geriatr Nurs. (2021) 42:635–42. doi: 10.1016/j.gerinurse.2021.03.015, PMID: 33823421

[64] Manuli A Maggio MG Tripoli D Gullì M Cannavò A la Rosa G. Patients' perspective and usability of innovation technology in a new rehabilitation pathway: an exploratory study in patients with multiple sclerosis. Mult Scler Relat Disord. (2020) 44:102312. doi: 10.1016/j.msard.2020.102312, PMID: 32585618

[65] Norman DA. Emotional design: Why we love (or hate) everyday things. New York: Basic Civitas Books (2004).

[66] De Bartolo D Morone G Lupo A. From paper to informatics: the post soft care-app, an easy-to-use and fast tool to help therapists identify unmet needs in stroke patients. Funct Neurol. (2018) 33:200–5. PMID: 30663966

[67] Mantovani E Zucchella C Bottiroli S Federico A Giugno R Sandrini G. Telemedicine and virtual reality for cognitive rehabilitation: a roadmap for the COVID-19 pandemic. Front Neurol. (2020) 11:926. doi: 10.3389/fneur.2020.00926, PMID: 33041963 PMC7522345

